# An Adaptive Calibration Framework for mVEP-Based Brain-Computer Interface

**DOI:** 10.1155/2018/9476432

**Published:** 2018-02-26

**Authors:** Teng Ma, Fali Li, Peiyang Li, Dezhong Yao, Yangsong Zhang, Peng Xu

**Affiliations:** ^1^Key Laboratory for Neuroinformation of Ministry of Education, School of Life Science and Technology, University of Electronic Science and Technology of China, Chengdu 610054, China; ^2^Department of Computer Science and Engineering, Henan Institute of Engineering, Zhengzhou 451191, China; ^3^Center for Information in Biomedicine, University of Electronic Science and Technology of China, Chengdu 610054, China; ^4^School of Computer Science and Technology, Southwest University of Science and Technology, Mianyang 621010, China

## Abstract

Electroencephalogram signals and the states of subjects are nonstationary. To track changing states effectively, an adaptive calibration framework is proposed for the brain-computer interface (BCI) with the motion-onset visual evoked potential (mVEP) as the control signal. The core of this framework is to update the training set adaptively for classifier training. The updating procedure consists of two operations, that is, adding new samples to the training set and removing old samples from the training set. In the proposed framework, a support vector machine (SVM) and fuzzy* C*-mean clustering (fCM) are combined to select the reliable samples for the training set from the blocks close to the current blocks to be classified. Because of the complementary information provided by SVM and fCM, they can guarantee the reliability of information fed into classifier training. The removing procedure will aim to remove those old samples recorded a relatively long time before current new blocks. These two operations could yield a new training set, which could be used to calibrate the classifier to track the changing state of the subjects. Experimental results demonstrate that the adaptive calibration framework is effective and efficient and it could improve the performance of online BCI systems.

## 1. Introduction

A brain-computer interface (BCI) provides an alternative communication and control channel between humans and the environment or devices by noninvasive [1–3] and invasive approaches [4]. For the noninvasive BCI, the scalp electroencephalogram (EEG) is the most-used modality to convey the user's intentions owing to its low cost and high portability for well-defined paradigms [5]. The well-designed paradigms in EEG-based BCIs include motor imagery [6, 7], steady-state visual evoked potentials (SSVEPs) [8–10], P300 event-related potentials [11, 12], and motion-onset visual evoked potential (mVEP) [13, 14]. Among these, mVEP is an important measure for studying the motion vision processing mechanisms of humans and animals. It has already been widely used in such fields as fundamental research and clinical diagnosis. For the neural mechanism of motion perception and physiological background of mVEP, the literature indicates that mVEP has advantages over other typical VEPs because of its large potential amplitude and minimal differences among and within the subjects [15]. These characteristics make mVEP more suitable in the application of BCIs. mVEP is evoked by the fast-moving visual stimulation and represents visual motion reactions of the middle temporal area and medial superior temporal area. mVEP typically contains three main peaks: a positive P1 peak with a latency of about 130 ms, a negative N2 peak with a latency of about 160–200 ms, and a positive P2 peak with a latency of approximately 240 ms [16]. N2 is the most prominent and stable component of the mVEP. The BCI group from Tsinghua University designed a stimulus paradigm to evoke mVEP and implemented it in a BCI system [14]. mVEP was successfully used to develop a spelling system similar to the P300 speller [17]. Because it does not need flashing stimulation or stimulation with sudden changes to evoke mVEP, the subjects are not prone to visual fatigue, which makes mVEP relatively more suitable for subjects in the training process. A common spatial pattern (CSP) algorithm has been proved to be a highly efficient feature extraction algorithm for BCI systems [18]. It aims to find directions (i.e., spatial filters) that maximize variance for one class and minimize variance for the other class at the same time [19]. The eigenvector processing by CSP is beneficial to the target recognition of BCI systems and improves the accuracy of the brain-computer interface system. In the current study, the CSP is used to extract features for the mVEP.

For a BCI system, we must collect a sufficient training dataset to train the classifier to implement online tasks. This procedure may be laborious and time consuming. To address this issue, a zero-training strategy and an automatic adapting mechanism have been explored [20–23]. The user's states could change during experiments due to unexpected environmental factors or internal physiological factor. In addition, EEG signals are highly subject-specific and vary considerably even between recording sessions of the same user within the same experimental paradigm [21–23]. Therefore, it is essential for an online system to track the changing states of subjects. In the traditional system, the classifier is usually trained before the online application [24–27]. When the subject's states change considerably from the previous states during the training stage, it is necessary to take some special measures, such as providing new data recorded from the subjects for the retraining and adjusting the classifier to track the subject's changing states. Some efforts on this topic have been tried [23, 28]. The main idea of those studies was to exploit the information in the previous sessions to calibrate the classifier. For example, Krauledat et al. proposed a method in which past sessions were used together to evaluate the prototype filters for the new session to calibrate the classifier. This approach does not need the training set and is the zero-training approach [23]. It seems that this approach only performs the calibration at the beginning stage of a new session. For an online system, when a session lasts for several hours, this method may be ineffective [1, 23, 29]. Therefore, calibration only at the beginning of a session may not be enough to capture the change of the subject's state that may occur during the experiment. It may be more meaningful to calibrate the classifier adaptively in the different phases of the experiment instead of just at certain specific periods. To this end, it is necessary to mine robustly the information hidden in the previous several blocks of data. The calibration performance largely depends on the reliability of the information represented by the previous blocks that could be used for classifier calibration. However, as for the practical online system, it may be very difficult or impossible to know exactly the tasks reflected by the sample; that is, we may not correctly label a sample with the classifier during the experiment.

The support vector machine (SVM) [30, 31] and fuzzy* C*-mean cluster (fCM) [32] are two different approaches, where the traditional SVM needs the training set for the supervised learning [30, 31]. It provides a link between the current block and the previously supervised classifier. fCM as a data-driven classifier does not need prior information as much as SVM for clustering [32], and it emphasizes the local clusters that the current samples form. Apparently, the different aspects of datasets can be reflected by these two different approaches, and the combination of them may provide more-flexible and more-reliable information about the samples.

In this paper, we propose an adaptive online calibration framework that was first used in mVEP-BCI system to calibrate the classifier that could track the changing states of the subjects. To fulfill this goal, the framework needs to adopt the new information in the latest samples and remove the information represented by the old samples, which were recorded a relatively long time previously. We combine SVM and fCM to select the reliable samples from the previous blocks and then clip the expanded training set to remove the old information represented by the old samples. With these operations, an updated training set could be generated and subsequently fed into the classifier for the retraining to track the subject's states. The performance of the framework was tested with the dataset from 11 subjects under the mVEP-based BCI paradigm. The results indicate the satisfactory effectiveness and efficiency of the proposed method.

The structure of this paper is as follows: The framework is introduced in Section 2, Section 3 presents the results when the adaptive calibration is used for the recorded dataset, and the discussion of the results and conclusions are given in Section 4.

## 2. Methods and Materials

### 2.1. The Traditional Training Protocol of a BCI Classifier

**Figure 1:**
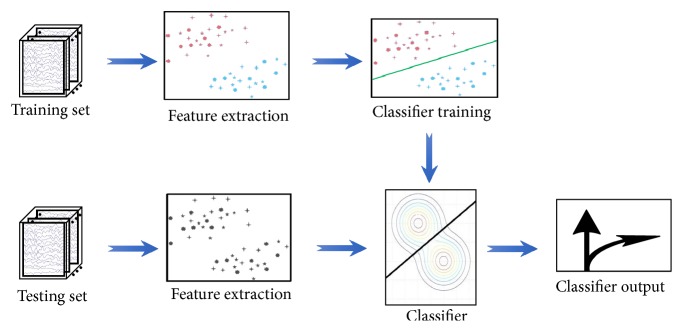
Classical classification flowchart for BCI tasks.

For most of the current BCI classifiers, training is usually implemented before the online experiment; that is, the training and test are not interactive [1, 33–35].  Figure 1 shows a flowchart for the traditional BCI classification used to classify BCI tasks.

The diagram reveals that the training set is usually fixed after the training procedure, and no new samples in the test set are adaptively updated into in the training set. For an online BCI system, the training set may be collected on different days, and the experiment may last for a relatively long time. Inevitably, the patterns according to the specific tasks may vary over time due to the nonstationarity and nonlinearity of EEG signals [33]. Therefore, the subject's state will surely change during the test stage compared with the state during the training stage. When the states are largely different in the two stages, the trained classifier may fail to decode the samples during new test sessions [21–23]. At this moment, the performance of the classifier will be inevitably lowered.

### 2.2. Adaptive Classifier Calibration Framework during the Experiment

Considering that the individual subject's state will vary during the experiment, it is beneficial to adapt the classifier to new data involving the varying states and to retrain it [21–23, 28]. To implement the adaptive mechanism, a direct approach is to integrate some new samples into the training set. However, it may be difficult to assign a reliable label to those new samples during the experiment. Obviously, once some unreliable samples are included in the training set, they may have a negative effect on the following classification performance [20, 31]. Therefore, it is vital to label the samples correctly and then add these reliable samples into the training set for further classifier calibration. The traditional SVM classification strategy may not yield satisfactory performance without recalibration using new samples when the bias between the training set and test set cannot be ignored.

SVM can provide the ability to discern how reliable an assigned label of a test sample is [31, 36]. It needs supervised training with training datasets; that is, the classification largely depends on the prior training data [31, 34, 36]. Unlike the SVM classifier, fCM is a kind of data-driven approach to classify the set without the training procedure [32]. The only prior information needed for fCM is the number of clusters, which is usually known for the BCI system. Apparently, fCM and SVM are two complementary approaches for classification, in that the former focuses on the similarity between the current sample and the previously labeled samples, whereas the latter aims at the current data distribution. Both can provide the probability (confidence interval) that indicates reliability for this classification. Certainly, the combination of these two methods can guarantee that the samples are classified with an accuracy that is more reliable than the single method. The following assumptions are considered in an adaptive BCI online system:

- The variance of subject's states will lead to classifier bias.
- The classifier calibration needs to be performed during a certain interval.
- The training set size cannot be too large for classifier training.

**Figure 2:**
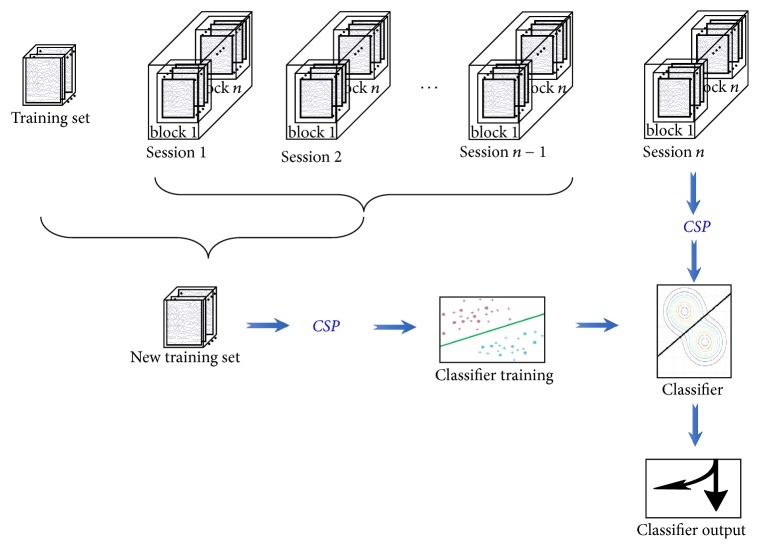
Framework for adaptive classifier calibration. Session *i* (1 ≤ *i* ≤ *n*) denotes the *i*th session consisting of several blocks, and each block includes several samples. Session *n* is the latest session to be classified. The existing training set and *n* − 1 sessions of new data were used to yield a new training set. CSP is used to extract the related features of the mVEP signal in the current work.

Based on these three assumptions, we proposed an adaptive framework for classifier calibration for a mVEP-based BCI system. The framework is shown in Figure 2.

**Figure 3:**
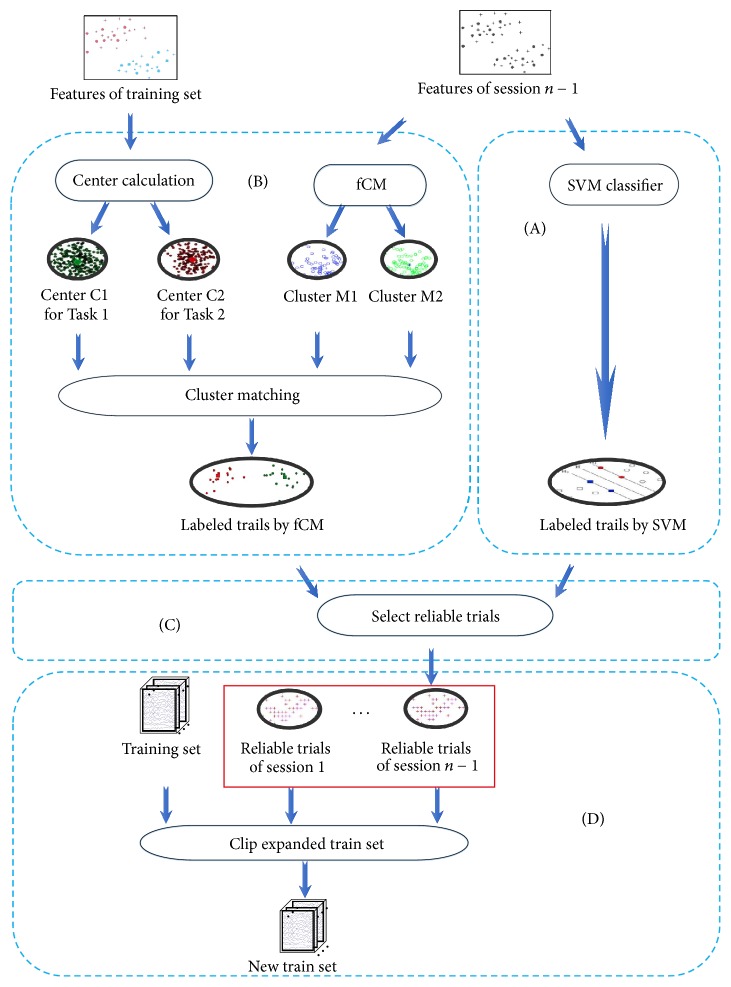
The procedure to generate the new training set.

The “new training set generation” process is the core of this framework and determines the performance of online BCI systems. If it is removed, the framework presented in Figure 2 becomes the traditional one. Considering the two-class task experiment, the detailed procedure of new training set generation is further revealed in Figure 3.

In Figure 3, the procedure for generating a new training set consists of Steps (A), (B), (C), and (D). For the adaptive classifier calibration, the procedure should include the new samples that can account for the subject's new state in the training set, and it should exclude the old samples that were recorded a relatively long time before current samples from the training set. The detailed implementation for the four subprocedures is elucidated as follows.

*Step (A)* (label samples with SVM). In this step, after the SVM classifier is trained by the old training set, the samples in session *n* − 1 are classified by this classifier. The output of this SVM classifier provides two kinds of information: the labels of samples and the probabilities denoting the reliability of those predicted labels [30, 36].

*Step (B)* (label samples with fCM). fCM is applied to the samples in session *n* − 1. Owing to the two-class classification task for mVEP data, fCM could classify the data into two clusters, M1 and M2, with cluster centers U1 and U2, respectively. As for clusters M1 and M2, we only know that these two clusters belong to different tasks and cannot exactly determine which labels (i.e., tasks) are assigned to M1 and M2. To label these two clusters, a matching technique is adopted. First, for the training dataset, the two centers C1 and C2 for the two tasks can be obtained by averaging the corresponding features. Then, the center U1 is compared with the centers C1 and C2. If U1 is much closer to C1, the samples in cluster M1 will be assigned with labels as the samples for Task 1 and samples in cluster M2 assigned with labels as samples for Task 2. Otherwise, samples in M1 and M2 are assigned with the labels as samples for Tasks 2 and 1, respectively. Besides the two clusters, fCM also generates a membership probability to indicate the reliability of each trial when it is assigned with the corresponding label [32].

*Step (C)* (select reliable trials). Based on the probabilities obtained with SVM and fCM that can indicate the classification reliability, we define a criterion to select reliable trials. Only the trials that have the same labels assigned by SVM and fCM can be regarded as potential candidates. Furthermore, we set an acceptance threshold *δ* (0 ≤ *δ* ≤ 1) for the selection operation. Let *P*_svm_(*i*) and *P*_fCM_(*i*) be the probabilities provided by SVM and fCM for the *i*th trial, respectively. If *P*_svm_(*i*) > *δ* and *P*_fCM_(*i*) > *δ*, then this trial will be selected as the reliable trial for succeeding classifier calibration.

*Step (D)* (clip the expanded training set). The subject's state may change over time; therefore, the samples in the training set recorded a relatively long time ago may have different characteristics and have a negative influence on classifier performance. Removing the redundant samples from the training set is necessary to include a fixed number of samples during the online experiment. This procedure is necessary for this adaptive classifier calibration framework. Without this clip procedure, the training set will grow quickly so that the training of the classifier will be unacceptable for the online system due to time-consuming training. Denoting the fixed number of the training samples as* M*, we label each sample with a time stamp in reverse time order. Specifically, the last added sample is labeled 1, the one before is labeled 2, and so on. When the size of the training set is larger than* M*, the clip procedure is implemented. We remove the samples that have a time stamp larger than* M* and keep the rest.

Considering that the subject's state will not greatly change in a relatively short time period, the calibration is performed at a certain time interval. In the current study, we adaptively updated the training set at a certain number of experiment blocks. Each block consisted of five trials that lasted for 1.5 s. With this framework, some new reliable samples could be integrated into the training set, while some old samples were excluded from the training set. In our work, SVM is used to classify the samples based on the expanded training set, and other classifiers, such as linear discriminate analysis (LDA) [33], Bayesian linear discriminate analysis (BLDA) [35], and kernel spectrum regression (KSR) [37], could be considered to replace SVM for classification.

### 2.3. Experimental Paradigm and Subjects

Eleven subjects (three females and eight males, age 23.6 ± 1.2 years) participated in the experiment. They had either normal vision or corrected-to-normal vision. The Institution Research Ethics Board of the University of Electronic Science and Technology of China approved the experimental protocol. All the subjects read and signed an informed consent form before they participated in the experiment.

**Figure 4:**
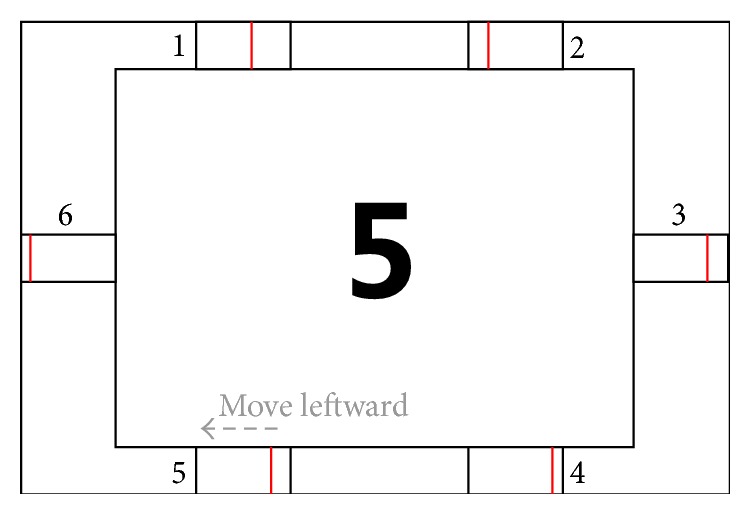
Graphical user interface for the offline data recording for mVEP-based BCI experiment. The number “5” in the center indicates the target button that subjects should gaze at. The red vertical line moves leftward with a random order in each of the six buttons to form the motion-onset stimulus.

A 14 in LCD monitor with a 1280 × 1024 resolution and 60 Hz refresh rate was used to present the visual stimulus graphical user interface (GUI) with a visual field of 30°  ×  19° on the screen, as shown in Figure 4. The six virtual buttons labeled with 1, 2, 3, 4, 5, and 6 were embedded in the GUI. Each button with a visual field of 4°  ×  2° was composed of a red vertical moving line and a vacant rectangle where the line existed.

**Figure 5:**
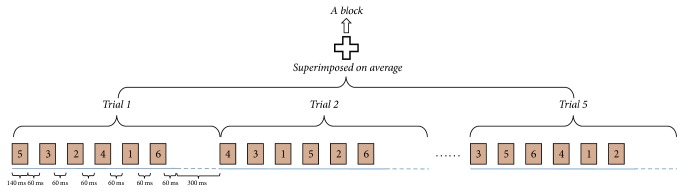
Timing scheme of the mVEP experiment. Each block contains five trials. In each trial, the motion stimulus appears in the virtual button for 140 ms. There is a 60 ms interval between two consecutive stimuli and a 300 ms interval between two consecutive trials.

For each button, the red line appeared and moved from the right side of the rectangle and disappeared at the leftmost side. The entire process formed a brief motion-onset stimulus and took 140 ms with a 60 ms interval between the consecutive move processes. Each motion-onset stimulus appears randomly in the corresponding virtual button, and all the stimuli appeared before others were repeated. A trial had six successive stimulus periods corresponding to the six buttons. Specifically, a trial included a series of six red vertical moving lines across each virtual button successively. Therefore, when there was a 300 ms interval between two trials, each trial lasted for 1.5 s, as shown in Figure 5. In addition, five trials formed a block, which lasted for 7.5 s.

In the experiment process, each subject was asked to focus on the button presented in the center of the GUI, where the random number appeared. And the subjects were required to calculate mentally the number of moving stimulus occurrences in the target button. A total of 72 blocks (including 360 trials) were collected for each subject in two separate sessions, and there is a 2 min interval for rest between the sessions. In the following process, the first session was used as the training set, and the second session was used as the test set. For the training set, we averaged five trials for each virtual button in each block. Then, we could get one target stimulation sample and five standard stimulation samples, where the sample in the current work refers to the 0.5 s long EEG recording corresponding to the stimulus. One standard stimulation sample was randomly selected and combined with the target stimulation sample as two samples. Thus, the data collected from each subject contain 36 pairs of samples to constitute the training set. For the test set, we also averaged the five trials for each virtual button, resulting in one target stimulation sample and five standard stimulation samples, and then we totally obtained six samples for one block in the test set. It was a binary classification problem for mVEP recognition. We needed to conduct six times the two classifications, and then we compared these output values to recognize the button at which the subject gazed. In this study, the accuracy was used to measure the subjects' performance, which is the ratio of the correctly classified blocks to the total blocks in the test set. It is obvious that the higher the recognition accuracy, the better the performance of mVEP-BCI.

By using a Symtop amplifier (Symtop Instrument, Beijing, China), eight Ag/AgCl electrodes (O3, O4, P3, P4, CP1, CP2, CP3, and CP4) from an extended 10–20 system were placed for EEG recordings. AFz electrode was adopted as reference. The EEG signals were sampled at 1000 Hz. There usually was noise contaminating the scalp-recorded EEG signals, and, in our work, those samples with absolute amplitude above the 50 *μ*v threshold were considered to be contaminated with strong artifacts and abandoned in the following analysis. Because the mVEP is usually distributed in the low-frequency band, EEG data were bandpass-filtered between 0.5 Hz and 10 Hz. Data between 150 ms and 300 ms were used to extract features with the CSP algorithm. The one pair of CSP filters was selected to filter the dataset. The log-variances of the spatially filtered data were fed into the classifier for training or test task recognition.

## 3. Results

This section details the performance evaluation of the proposed approach under various conditions based on the accuracy and information transfer rate. The accuracy is defined as the ratio of the number of correctly recognized targets to the number of targets overall. Besides accuracy, the corresponding information transfer rate (ITR) is another standard criterion to measure the BCI performance. Generally, ITR is defined as

(1) $$\begin{array}{c} ITR=\frac{\left({log}_{2}^{N}+P\;{log}_{2}^{P}+\left(1-P\right){log}_{2}^{\left[\left(1-p\right)/\left(N-1\right)\right]}\right)}{T}, \end{array}$$

where *N* is the number of selectable items, *P* is the selection accuracy, and *T* is the average time in seconds for finishing one selection.

### 3.1. Effect of the Calibration Interval

As the subject's state may change during certain intervals, in this section, the effect of the calibration interval on the classifier performance is explored. Specifically, we study the performance of the classifier when it is calibrated with different numbers of blocks.  Table 1 lists the accuracies and ITRs when the classifier is calibrated for every four, six, and nine blocks, respectively. For the different calibration intervals, the training set size is fixed at 250 samples, and the threshold for reliable sample selection is 0.75. The accuracies and ITRs in Table 1 are the overall accuracies and ITRs for the total 36 test blocks. The test set is thus divided into several segments that have the same number of samples as the adopted calibration interval. The samples in the current segment are classified using the classifier calibrated with samples in previous segments. The original SVM classifier uses the total training set of 36 pairs of samples. In the calibration framework, the samples in the previous segment are used to update the training set, and the redundant samples are dynamically removed to keep a fixed number of training samples for adaptive calibration of classifiers. The same default parameters were used for SVM classifiers in the calibration and classification stages for relatively fair comparison. The results of a single SVM calibration and single fCM calibration under different calibration intervals are shown in Tables 2 and 3. We find that the combination of SVM and fCM yields better performance with higher accuracy and ITR than a single method. Additionally, the performance obtained by the combination strategy is significantly higher than the performance obtained by the method without adaptive calibration under three interval conditions.

### 3.2. Effect of the Threshold for Reliable Sample Selection

In this subsection, the influence of the threshold for reliable sample selection on the calibration performance is explored. Five values, that is, 0.6, 0.65, 0.7, 0.75, and 0.8, were tested. The calibration was performed every four blocks.  Table 4 gives the overall accuracies and ITRs when different thresholds were used. The results of a single SVM calibration and single fCM calibration under different thresholds are also shown in Tables 5 and 6. We observe that the calibration with the combination of fCM and SVM shown in Table 4 provides better performance (i.e., higher accuracy and ITR) with a significant difference compared with single fCM or SVM calibration at each threshold. In addition, the threshold of 0.75 provides the best performance. These results further confirm that the proposed framework combining fCM and SVM is feasible and effective and it is superior to the two methods when they were implemented independently.

## 4. Discussion and Conclusion

The calibration of the classifier is an open issue for BCI online systems, and the application of the information contained in the new samples is one feasible solution to this issue [20, 22, 28]. However, for the practical online system, it is impossible or difficult to label the samples as indisputably correct. To address this problem, we proposed an adaptive classifier calibration framework. In this framework, we labeled the samples according to the outputs of SVM and fCM, and we chose the reliable samples to update the training set that was used to recalibrate the classifier. Moreover, two parameters, that is, the calibration interval and the threshold for reliable sample selection, were studied. We systematically tested the effects of the two parameters on the classifier performance.

As shown in Table 1, when the calibration interval varies, the calibration effect for the classifier is different. Among the three different intervals tested, the interval including four blocks demonstrates the best performance, and the average accuracy is 88.4% and the average ITR is 14.7. However, compared with the original SVM approach without calibration, whatever the three calibration intervals the calibration approach adopts, the classification accuracy is significantly improved. The average classification accuracies of four-block interval, six-block interval, and nine-block interval are improved from 85.6% to 88.4%, 88.2%, and 87.4%, accompanied by the improved ITRs from 13.5 to 14.7, 14.6, and 14.2, respectively. For a practical online system, there is no doubt that the subject's state may change during the experiment, but it may not be necessary to calibrate the classifier for each block or at a short interval. If the calibration is frequently adapted, the efficiency of the online system may be lowered due to the extra calculation involved. Moreover, the subject's state within a certain duration will be kept relatively stable, so the feasible way is to calibrate the classifier after a certain long period. However, the calibration interval cannot be too large, or it may fail to adapt the classifier to track the subject's state on-time.

As shown in Table 2, when we only adopt the outputs of SVM to find the reliable samples to update the training set, the average performances (i.e., the accuracy and ITR) of the three calibration intervals are 85.1% (13.3 bits), 85.1% (13.4 bits), and 84.6% (13.2 bits), respectively. Compared with the original SVM approach without classifier calibration, the average performance is not improved. We can see similar results in Table 3. When we only use the outputs of fCM to find the reliable samples to update the training set, the average performances of the three calibration intervals are 85.4% (13.5 bits), 85.1% (13.3 bits), and 84.3% (13.0 bits), respectively. Obviously, the average performance evaluated with accuracy and ITR has not yet been improved. For each subject, compared with the method of combining SVM and fCM to perform the classifier calibration, most of the subjects' performance becomes worse for the single method. We can see that the adoption of a single fCM or SVM for calibration is not sufficiently effective. The method using the combination of SVM with fCM is superior to single SVM or single fCM and may provide more-reliable information about the new samples.

The reliability of the selected sample is crucial for the classifier calibration. The performance improvement of the calibration approach is mainly due to the use of the information in the new samples to retrain the classifier. In this framework, the combination of two different approaches can reflect the different aspects of samples to mine the information hidden in the new samples. As shown in Table 4, when the threshold is varied as 0.60, 0.65, 0.7, 0.75, and 0.80, the calibration approach gives the classification with average performances of 87.9% (14.4 bits), 87.6% (14.3 bits), 88.1% (14.5 bits), 88.4% (14.7 bits), and 87.1% (14.2 bits), respectively, compared with the baseline 85.6% (13.5 bits) of the original SVM classifier. The results show that the selection of the threshold influences the performance of the calibration approach, and the best accuracy of 88.4% and highest ITR of 14.7 bits were achieved when the threshold was 0.75. The threshold serves as a filter to differentiate the reliable samples and unreliable samples, and a larger threshold will facilitate selection of the more reliable samples. For the lower thresholds, these thresholds could not guarantee the selection of the samples with high-confidence probability; that is, some incorrectly labeled samples could be added to the training set. Obviously, those mislabeled samples may give incorrect information for classifier calibration (training). Therefore, the performances of thresholds that are 0.60 and 0.65 are lower than those of 0.75. When the threshold becomes too large, few reliable samples can be selected in one calibration interval, and the small number of new samples in the training set will not be enough or effective to calibrate the classifier, which may be the main reason that the performance of 0.8 is not compared with that of 0.75. As shown in Tables 5 and 6, we found that when only SVM or fCM was adopted to perform the classifier calibration, the average performance of the five thresholds was not obviously improved compared with the original SVM approach. For each subject, compared with the combination of SVM and fCM to perform the classifier calibration, most of the subjects' performance becomes worse when the single SVM classifier is used for calibration. Therefore, these results further confirmed that combination of SVM and fCM to perform the classifier calibration could provide better performance to find reliable samples.

**Figure 6:**
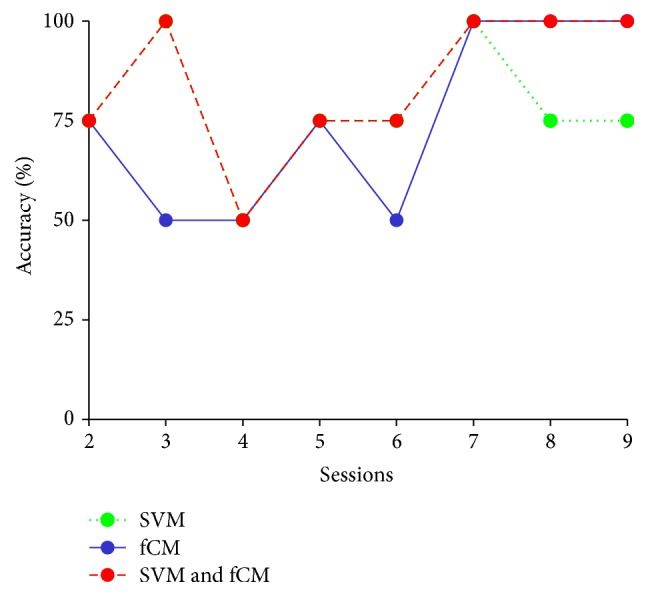
The identification accuracy of Subject 1 in each session among the three calibration methods.

In summary, Tables 2, 3, 5, and 6 consistently revealed that when calibration was performed by the single SVM or fCM, it did not show obvious improvement, whereas when SVM and fCM were combined to calibrate the classifier, higher performance with higher accuracy and ITR was exhibited. The difference is attributed to the enhanced ability of the proposed approach to capture reliable information from the testing set. To further reveal this difference, we analyze the different effects when using the combination of SVM and fCM, single SVM, and single fCM to calibrate the classifier for each session.  Table 7 shows the correct number of samples, the total number of samples updated into the training set, and the ratio of the two kinds of samples from a representative subject (Subject 1) by adoption of the three methods, respectively.  Figure 6 shows the corresponding identification accuracy for each session. The threshold for reliable sample selection was set at 0.75, and the calibration interval was set at four blocks.

From Table 7, we observe that the ratio of screened samples with correct labels to total screened samples is higher when using the combination of SVM and fCM than with SVM or fCM to perform the classifier calibration. It is obvious that the combined calibration method could provide a training set with more-reliable new samples. Similarly, from Figure 6, we see the differences of identification accuracy among the three calibration methods for each session. The combined method of SVM and fCM is overall better than the other two methods, which is attributed to the higher sample ratio of corrected labels to be added to the training set by the combined approach. SVM and fCM are two different approaches to handle the unlabeled samples: SVM needs the training set to train the classifier, while fCM is a kind of data-driven approach that can classify the dataset without the need to have the training set [31, 32, 34, 37]. The reliability of the information added to the training set will determine the algorithm's performance [22, 23, 31]. In this work, the outputs of SVM and fCM were combined to find the reliable samples to update the training set, which may make the classifier calibration more robust.

After new samples were added to the training set, the clip technique was used to remove the old samples that were recorded a long time before current blocks. This technique facilitates the BCI online system in two ways. First, the removal of the old samples will be helpful to track the subject's state, because those old samples may represent the different subject's stage from the current stage, and their utilization for training could distort the classifier. Second, the online system requires a not-too-large training set for effective training [28, 33], and the clip technique can keep the size of the training set fixed.

The result in this work is offline analysis for mVEP-BCI data of our lab. We will transplant this framework to our BCI online system in the future. Moreover, SVM is used in the current version and other classifiers, such as LDA [33, 38], BLDA [35], and KSR [37], could be adopted. To have a relatively fair comparison, the default setups of SVM are used in the current work. If the SVM parameters are optimized with a technique like grid searching [36], the performances for both approaches may be further improved, but we think the relative performance between them will be similar to that reported here. Our framework aims to calibrate the classifier adaptively during the long experiment duration, and it still needs certain training procedures to train the classifier initially; that is, our framework is not the zero-training online system [23].The data-driven fCM can classify the dataset without the training procedure, and we will study the possibility of extending this system to zero training. The adapting strategy used in the framework assumes that the subject's state will gradually change during the experiment (i.e., it is possible to track the state's change). If the subject's state varies abruptly, our calibration framework may fail to track this change. Under this special condition, it may be necessary to provide the subject with a new training session for totally new classifier training.

In all, the above results demonstrate that the proposed adaptive calibration framework, which was first used in the mVEP-BCI system, can improve the BCI classifier performance. The core of the proposed framework is adaptively updating the training set and recalibrating the classifier. One way of updating is to add the novel information that can reflect the subject's current state to the training set, and another way is to remove the old information from the training set. By merging information in the new samples with the training set, the classifier could track the changes of the subject's states. The feasibility and effectiveness were verified by the real offline EEG data. Accordingly, the proposed framework is a promising methodology for adaptively improving the mVEP-based BCI system, and it could be generalized to other BCI modalities.

## Figures and Tables

**Table 1:** Performance of both calibrations when the classifier is calibrated with a different number of blocks.

Subjects	Adaptive calibrationby SVM and fCM (accuracy (%)/ITR)			SVM
	4	6	9	
S1	86.1/13.4	81.5/11.7	83.3/12.4	83.3/12.4
S2	94.4/17.1	94.4/17.1	91.7/15.8	91.7/15.8
S3	69.4/7.9	72.2/8.7	72.2/8.7	66.7/7.2
S4	94.4/17.1	97.2/18.7	94.4/17.1	91.7/15.8
S5	77.8/10.4	80.6/11.4	77.8/10.4	75/9.5
S6	91.7/15.8	88.9/14.6	88.9/14.6	88.9/14.6
S7	94.4/17.1	94.4/17.1	91.7/15.8	91.7/15.8
S8	94.4/17.1	94.4/17.1	91.7/15.8	88.9/14.6
S9	94.4/17.1	94.4/17.1	94.4/17.1	94.4/17.1
S10	83.3/12.4	77.8/10.4	80.6/11.4	77.8/10.4
S11	91.7/15.8	94.4/17.1	94.4/17.1	91.7/15.8

Mean ± std	88.4 ± 8.0^*∗*^/14.7 ± 3.1^*∗*^	88.2 ± 8.2^*∗*^*/*14.6 ± 3.3^*∗*^	87.4 ± 7.3^*∗*^*/*14.2 ± 2.8^*∗*^	85.6 ± 8.9*/*13.5 ± 3.1

*∗* denotes that the adaptive calibration method results are significantly higher than those of SVM approach (*p* < 0.05, paired *t*-test). In each column, the left and right values of “/” denote the accuracies and ITRs of subjects, respectively.

**Table 2:** Performance of single SVM calibration when the classifier is calibrated with different numbers of blocks.

Subjects	Adaptive calibration by SVM (accuracy (%)/ITR)			SVM
	4	6	9	
S1	80.6/11.4	80.6/11.4	83.3/12.4	83.3/12.4
S2	94.4/17.1	91.7/15.8	91.7/15.8	91.7/15.8
S3	63.9/6.4	66.7/7.2	63.9/6.4	66.7/7.2
S4	88.9/14.6	91.7/15.8	88.9/14.6	91.7/15.8
S5	75/9.5	72.2/8.7	75/9.5	75/9.5
S6	88.9/14.6	86.1/13.4	88.9/14.6	88.9/14.6
S7	91.7/15.8	88.9/14.6	91.7/15.8	91.7/15.8
S8	88.9/14.6	91.7/15.8	88.9/14.6	88.9/14.6
S9	91.7/15.8	94.4/17.1	94.4/17.1	94.4/17.1
S10	83.3/12.4	80.6/11.4	75/9.5	77.8/10.4
S11	88.9/14.6	91.7/15.8	88.9/14.6	91.7/15.8

Mean ± std	85.1 ± 8.5/13.3 ± 3.0	85.1 ± 8.6/13.4 ± 3.1	84.6 ± 9.0/13.2 ± 3.2	85.6 ± 8.9/13.5 ± 3.1

**Table 3:** Performance of single calibration when the classifier is calibrated with different numbers of blocks.

Subjects	Adaptive calibration by fCM (accuracy (%)/ITR)			SVM
	4	6	9	
S1	77.8/10.4	83.3/12.4	77.8/10.4	83.3/12.4
S2	91.7/15.8	91.7/15.8	88.9/14.6	91.7/15.8
S3	66.7/7.2	69.4/7.9	66.7/7.2	66.7/7.2
S4	91.7/15.8	88.9/14.6	88.9/14.6	91.7/15.8
S5	77.8/10.4	75/9.5	75/9.5	75/9.5
S6	94.4/17.1	91.7/15.8	88.9/14.6	88.9/14.6
S7	86.1/13.4	88.9/14.6	86.1/13.4	91.7/15.8
S8	88.9/14.6	86.1/13.4	88.9/14.6	88.9/14.6
S9	91.7/15.8	94.4/17.1	94.4/17.1	94.4/17.1
S10	77.8/10.4	75/9.5	77.8/10.4	77.8/10.4
S11	94.4/17.1	91.7/15.8	94.4/17.1	91.7/15.8

Mean ± std	85.4 ± 8.6/13.5 ± 3.2	85.1 ± 8.0/13.3 ± 2.9	84.3 ± 8.4/13.0 ± 3.1	85.6 ± 8.9/13.5 ± 3.1

**Table 4:** Performance of both calibrations when the threshold for reliable sample selection takes different values.

Subjects	Adaptive calibration by SVM and fCM (accuracy (%)/ITR)					SVM
	0.6	0.65	0.7	0.75	0.8	
S1	83.3/12.4	83.3/12.4	86.1/13.4	86.1/13.4	83.3/12.4	83.3/12.4
S2	94.4/17.1	91.7/15.8	94.4/17.1	94.4/17.1	94.4/17.1	91.7/15.8
S3	72.2/8.7	69.4/7.9	69.4/7.9	69.4/7.9	66.7/7.2	66.7/7.2
S4	94.4/17.1	91.7/15.8	91.7/15.8	94.4/17.1	94.4/17.1	91.7/15.8
S5	80.6/11.4	80.6/11.4	80.6/11.4	77.8/10.4	77.8/10.4	75/9.5
S6	88.9/14.6	88.9/14.6	91.7/15.8	91.7/15.8	91.7/15.8	88.9/14.6
S7	88.9/14.6	94.4/17.1	91.7/15.8	94.4/17.1	91.7/15.8	91.7/15.8
S8	91.7/15.8	91.7/15.8	94.4/17.1	94.4/17.1	91.7/15.8	88.9/14.6
S9	97.2/18.7	94.4/17.1	94.4/17.1	94.4/17.1	94.4/17.1	94.4/17.1
S10	80.6/11.4	83.3/12.4	80.6/11.4	83.3/12.4	80.6/11.4	77.8/10.4
S11	94.4/17.1	94.4/17.1	94.4/17.1	91.7/15.8	91.7/15.8	91.7/15.8

Mean ± std	87.9 ± 7.8^*∗*^/14.4 ± 3.0^*∗*^	87.6 ± 7.8^*∗*^/14.3 ± 2.8^*∗*^	88.1 ± 8.1^*∗*^/14.5 ± 3.0^*∗*^	88.4 ± 8.4^*∗*^/14.7 ± 3.1^*∗*^	87.1 ± 9.0^*∗*^/14.2 ± 3.2^*∗*^	85.6 ± 8.9/13.5 ± 3.1

*∗* denotes that the adaptive calibration method results are significantly higher than those of SVM approach (*p* < 0.05, paired *t*-test).

**Table 5:** Performance of single SVM calibration when the threshold for reliable sample selection takes different values.

Subjects	Adaptive calibration by SVM (accuracy (%)/ITR)					SVM
	0.6	0.65	0.7	0.75	0.8	
S1	80.6/11.4	83.3/12.4	80.6/11.4	80.6/11.4	80.6/11.4	83.3/12.4
S2	94.4/17.1	94.4/17.1	91.7/15.8	94.4/17.1	94.4/17.1	91.7/15.8
S3	63.9/6.4	66.7/7.2	66.7/7.2	63.9/6.4	61.1/5.7	66.7/7.2
S4	86.1/13.4	88.9/14.6	88.9/14.6	88.9/14.6	91.7/15.8	91.7/15.8
S5	77.8/10.4	80.6/11.4	75/9.5	75/9.5	77.8/10.4	75/9.5
S6	88.9/14.6	86.1/13.4	88.9/14.6	88.9/14.6	88.9/14.6	88.9/14.6
S7	91.7/15.8	91.7/15.8	91.7/15.8	91.7/15.8	91.7/15.8	91.7/15.8
S8	86.1/13.4	88.9/14.6	88.9/14.6	88.9/14.6	88.9/14.6	88.9/14.6
S9	94.4/17.1	91.7/15.8	91.7/15.8	91.7/15.8	91.7/15.8	94.4/17.1
S10	77.8/10.4	80.6/11.4	83.3/12.4	83.3/12.4	80.6/11.4	77.8/10.4
S11	91.7/15.8	91.7/15.8	91.7/15.8	88.9/14.6	88.9/14.6	91.7/15.8

Mean ± std	84.9 ± 9.2/13.3 ± 3.2	85.9 ± 7.9/13.6 ± 2.7	85.4 ± 8.3/13.4 ± 2.8	85.1 ± 9.0/13.3 ± 3.0	85.1 ± 9.6/13.4 ± 3.2	85.6 ± 8.9/13.5 ± 3.1

**Table 6:** Performance of single fCM calibration when the threshold for reliable sample selection takes different values.

Subjects	Adaptive calibration by fCM (accuracy (%)/ITR)					SVM
	0.6	0.65	0.70	0.75	0.8	
S1	83.3/12.4	80.6/11.4	77.8/10.4	77.8/10.4	80.6/11.4	83.3/12.4
S2	91.7/15.8	91.7/15.8	94.4/17.1	91.7/15.8	94.4/17.1	91.7/15.8
S3	63.9/6.4	66.7/7.2	66.7/7.2	66.7/7.2	72.2/8.7	66.7/7.2
S4	91.7/15.8	91.7/15.8	88.9/14.6	91.7/15.8	88.9/14.6	91.7/15.8
S5	77.8/10.4	75/9.5	77.8/10.4	77.8/10.4	75/9.5	75/9.5
S6	91.7/15.8	91.7/15.8	91.7/15.8	94.4/17.1	86.1/13.4	88.9/14.6
S7	88.9/14.6	88.9/14.6	88.9/14.6	86.1/13.4	86.1/13.4	91.7/15.8
S8	88.9/14.6	86.1/13.4	86.1/13.4	88.9/14.6	88.9/14.6	88.9/14.6
S9	94.4/17.1	91.7/15.8	91.7/15.8	91.7/15.8	91.7/15.8	94.4/17.1
S10	77.8/10.4	80.6/11.4	77.8/10.4	77.8/10.4	77.8/10.4	77.8/10.4
S11	91.7/15.8	91.7/15.8	94.4/17.1	94.4/17.1	91.7/15.8	91.7/15.8

Mean ± std	85.6 ± 9.2/13.6 ± 3.1	85.1 ± 8.5/13.3 ± 2.9	85.1 ± 8.9/13.3 ± 3.1	85.4 ± 9.0/13.5 ± 3.2	84.9 ± 7.4/13.2 ± 2.7	85.6 ± 8.9/13.5 ± 3.1

**Table 7:** Number of samples updated and ratio of correctly recognized samples by three methods.

Session	Adaptive calibration by SVM			Adaptive calibration by fCM			Fusion adaptive calibration		
	A	B	C	A	B	C	A	B	C
2	13	15	0.867	4	6	0.667	3	4	0.750
3	9	14	0.643	3	5	0.600	4	6	0.667
4	10	13	0.769	2	4	0.500	3	4	0.750
5	11	14	0.786	3	5	0.600	4	5	0.800
6	13	15	0.867	5	6	0.833	4	4	1.000
7	10	13	0.769	4	5	0.800	3	4	0.750
8	9	11	0.818	6	6	1.000	3	3	1.000
9	12	14	0.857	7	7	1.000	3	3	1.000

Sum	87	109	0.798	34	44	0.773	27	33	0.818

A and B denote the number of correctly labeled samples and the total number of samples updated into the training set, and C denotes the ratio of A and B.
